# Fluid Shifts Induced by Physical Therapy in Lower Limb Lymphedema Patients

**DOI:** 10.3390/jcm9113678

**Published:** 2020-11-16

**Authors:** Bianca Brix, Gert Apich, Andreas Roessler, Christian Ure, Karin Schmid-Zalaudek, Helmut Hinghofer-Szalkay, Nandu Goswami

**Affiliations:** 1 Physiology Division, Otto Loewi Research Center, Gravitational Physiology and Medicine Research Unit, Medical University of Graz, 8036 Graz, Austria; bianca.brix@medunigraz.at (B.B.); andreas.roessler@medunigraz.at (A.R.); karin.schmid@medunigraz.at (K.S.-Z.); helmut.hinghofer@medunigraz.at (H.H.-S.); 2 Physical Medicine and General Rehabilitation Department, KABEG, Wolfsberg Site, 9400 Wolfsberg, Austria; gert.apich@kabeg.at; 3 Wolfsberg Clinical Center for Lymphatic Disorders, Wolfsberg State Hospital, KABEG, 9400 Wolfsberg, Austria; christian.ure@kabeg.at

**Keywords:** lymph, lymphatic flow, complete decongestive therapy, lymphatic drainage

## Abstract

Complete decongestive therapy (CDT), a physical therapy including manual lymphatic drainage (MLD) and compression bandaging, is aimed at mobilizing fluid and reducing limb volume in lymphedema patients. Details of fluid shifts occurring in response to CDT are currently not well studied. Therefore, we investigated fluid shifts before, during and after CDT. Thirteen patients (3 males and 10 females, aged 57 ± 8.0 years, 167.2 ± 8.3 cm height, 91.0 ± 23.4 kg weight) diagnosed with stage II leg lymphedema participated. Leg volume, limb and whole-body fluid composition (total body water (limbTBW/%TBW), extracellular (limbECF/%ECF) and intracellular (limbICF/%ICF fluid), as well as ECF/ICF and limbECF/limbICF ratios were determined using perometry and bioelectrical impedance spectroscopy. Plasma volume, proteins, osmolality, oncotic pressure and electrolytes were assessed. Leg volume (*p* < 0.001), limbECF (*p* = 0.041), limbICF (*p* = 0.005) and limbECF/limbICF decreased over CDT. Total leg volume and limbTBW were correlated (r = 0.635). %TBW (*p* = 0.001) and %ECF (*p* = 0.007) decreased over time. The maximum effects were seen within one week of CDT. LimbICF (*p* = 0.017), %TBW (*p* = 0.009) and %ICF (*p* = 0.003) increased post-MLD, whereas ECF/ICF decreased due to MLD. Plasma volume increased by 1.5% post-MLD, as well as albumin and the albumin-to-globulin ratio (*p* = 0.005 and *p* = 0.049, respectively). Our results indicate that physical therapy leads to fluid shifts in lymphedema patients, with the greatest effects occurring within one week of therapy. Fluid shifts due to physical therapy were also reflected in increased plasma volume and plasma protein concentrations. Perometry, in contrast to bioelectrical impedance analysis, does not seem to be sensitive enough to detect small fluid changes caused by manual lymphatic drainage.

## 1. Introduction

The lymphatic vasculature is a second network of vessels, adjacent to the blood vasculature. It plays a crucial role in normal physiology [1,2]. The dynamics of blood pressure and oncotic pressure drive a continuous filtration of blood plasma from blood capillaries into the interstitial space. Excess fluid and macromolecules are taken up from the interstitial space into the initial lymphatics through a permeable endothelial cell layer [3]. The blind-ended lymphatic capillaries take up lymphatic fluid, which is then transported through a hierarchy of lymphatic vessels throughout the body. Ultimately, lymphatic fluid is returned back into the vessels of the cardiovascular system via the thoracic duct [1]. Initial protein concentration of the lymphatic fluid is around 3–4%. Once it reaches the blood stream, protein concentration is believed to be higher. It reaches levels similar to blood plasma (≈6%) [4]. It is estimated that approximately eight liters of lymphatic fluid are generated every day. Due to reabsorption processes within the lymph nodes, the post-nodal flow rate is about four liters per day [5]. Any dysfunction within the lymphatic vasculature can lead to the accumulation of fluid within the tissue. This occurs due to an imbalance of plasma filtration and transport capacity of the vessels [2,6], resulting in different pathological conditions, such as lymphedema.

Lymphedema is characterized by a chronic, progressive and disfiguring form of regional swelling. The disease is usually diagnosed in peripheral extremities [2,6]. The amount of fluid accumulated can reach up to 5–15 L [7]. It requires continuous treatment including meticulous care on behalf of the patient, non-invasive therapy options, such as physical therapy, and surgery [8,9]. However, no definite cure is currently available. Physical therapy in the form of complete decongestive therapy (CDT) can assist in reducing symptoms. CDT lasts for several weeks and is a multicomponent therapy program with the primary aim to reduce volume and to prevent disease progression [8,10,11]. This physical therapy consists of manual lymphatic drainage (MLD), bandaging and compression of the affected tissue. Additionally, physical exercises and educational seminars are part of the treatment. CDT has been reported to increase lymphatic outflow [12] and lead to fluid mobilization [13,14]. However, limited knowledge is available on the amount of fluid being shifted during therapy, where this fluid is shifted to and its systemic effects. As far as we are aware, no previous study has systematically investigated fluid shifts due to physical therapy in lymphedema patients and how these are reflected in plasma volume changes as well as changes in plasma component concentrations. Therefore, this study investigated fluid shifts, assessed via perometry and bioelectrical impedance analysis, and their effect on blood plasma components before, during and after CDT. We hypothesized that leg volume as well as extracellular and total fluid in the affected extremities will reduce over CDT as well as due to MLD. Furthermore, as the lymphatic system drains into the blood vessels, we assessed changes in plasma volume and plasma components such as proteins (albumin, globulin and total protein) and electrolytes (sodium, chloride and potassium). We hypothesized that the fluid shifts during therapy would also be reflected in increased plasma volume post-therapy. As indicated above, protein concentration of the returned lymph fluid is estimated to be at the same level as blood plasma [4]. Therefore, we hypothesized that no changes in plasma protein concentrations will occur.

## 2. Experimental Section

This study was conducted at the Clinical Center for Lymphatic Disorders, State Hospital, Wolfsberg, Austria. All collected data were analyzed at the Medical University of Graz. Both ethics committees—Medical University Graz, Austria (EK: 29-090 ex 16/17) and Carinthia (EK: A 03/17)—approved the study. Good clinical practices and the WMA Declaration of Helsinki (2013) were followed throughout the study. Each patient received detailed information about the study protocol before giving written consent.

### 2.1. Patients

Patients with stage II (in accordance with the 2020 Consensus Document of the International Society of Lymphology [15]) primary or secondary lower limb lymphedema were included in this study. Patients with any signs of mental disorders, those with histories of cardiovascular diseases, syncope or alcoholism as well as those on specific medications were excluded from participation. Patients who received complete decongestive therapy within one year prior to this study were not included in this study. Pregnant women and patients taking part in any other clinical trial during the same period were excluded. Based on previously reported results [16], a strong effect size was to be expected (f = 0.5). The a priori sample size calculation for the repeated measures ANOVA, considering α = 0.05 and a power = 0.90, resulted in an estimated total sample size of 13 patients.

### 2.2. Complete Decongestive Therapy Protocol

The complete decongestive therapy protocol used in this study strictly followed the recommendations by Döller (2013) [10,17] and was performed by specialized physiotherapists at the Clinical Center for Lymphatic Disorders, Wolfsberg. Complete decongestive therapy consisted of several phases, which were (a) manual lymphatic drainage (MLD) and (b) the application of compression bandages. Patients received MLD for 30 min each day from Monday to Friday. MLD comprised of distinct hand movements, such as rhythmic, flowing, stirring and scrubbing motions applied with low pressure (30–40 mmHg), also including the stretching of the skin subcutis. These slow gestures were performed to simulate the spontaneous frequency of the lymphangion, which is around 10 contractions per minute. MLD started in the healthy tissue areas and was then extended to the lymphedematous tissue. Compression garments were applied to the affected limbs directly after MLD [10]. Additionally, the patients performed physical exercises such as ergometry, walking and/or gymnastics whilst wearing those bandages the entire day and overnight. Finally, educational seminars on self-care, nutrition and skin care as well as psychological assistance were part of CDT, as well.

### 2.3. Data Collection

**Figure 1:**
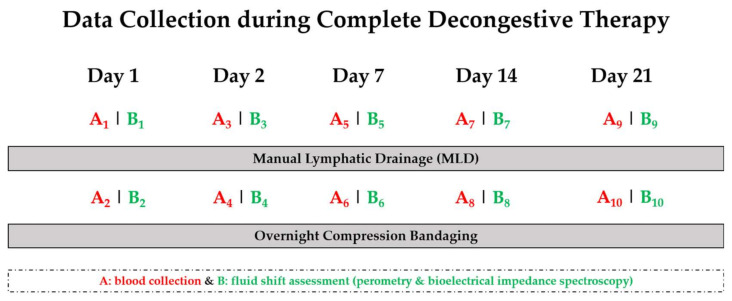
Overview of the study design. Blood collection (**red**) and fluid shift assessments (**green**) were performed on day 1, 2, 7, 14 and 21 of complete decongestive therapy (CDT), before and after manual lymphatic drainage (MLD).

Data collection took place in the morning, between 08:00 a.m. and 11:00 p.m., in a dimly lit quiet room. The temperature was set between 22–25 °C and the humidity remained constant between 50–55%. Fluid shift assessments (which were carried out using perometry and bioelectrical impedance spectroscopy, BIS as well as in the blood) to determine time-course effects over physical therapy were performed on the following days of CDT: day 1, day 2, day 7, day 14 and day 21. Additionally, in order to investigate the effect of MLD, all assessments were performed directly before and after 30 min of MLD on each of the indicated days (Figure 1).

### 2.4. Blood Collection

Patients were asked to remain seated for blood collection. Fifty milliliters of venous blood was collected from the ante-cubital vein. EDTA blood samples were immediately cooled and then centrifuged (1500× *g*, 15 min, 4 °C). Plasma was stored in aliquots at −80 °C until further measurements. Hematocrit (Hct) was determined in duplicates (10 min at 10,000 rpm). Plasma density (PD) was assessed using a high-precision mass densitometry device (model 602 M, Paar KG, Graz, Austria) on 0.2 mL samples via the mechanical oscillator technique. Density determinations were performed at 37.00 ± 0.02 °C controlled by an ultrathermostat (Hetofrig, Faaborg, Denmark). Mass density (FD) was calculated from the corresponding plasma density (PD) and hematocrit (Hct) values. For further details, please see Hinghofer-Szalkay and colleagues (2008 and 2011) [18,19]. Protein concentrations, osmolality, oncotic pressure and electrolytes (sodium, chloride and potassium) were all determined via routine machines used at the clinical chemistry laboratory.

### 2.5. Assessment of Volume and Fluid Mobilization

For limb volume assessment of the legs, a perometer type 550 T together with the PeroPlus 2000 software (Pero-System Messgeräte, Wuppertal, Germany) was used. Assessment of fluid composition was performed using the SFB7 device (ImpediMed Ltd., Brisbane, Australia) and the manufacturer’s software (Bioimp 5.5.0.1., ImpediMed Ltd., Brisbane, Australia). Patients were asked to empty their bladder prior to the measurement. Electrodes were positioned on previously disinfected skin of the patients in supine position. Total body composition analysis as well as segmental measurements in the lower limbs were performed [20,21]. Percentages of total body water (%TWB), extracellular fluid (%ECF) and intracellular fluid (%ICF) to the individual patient’s body weight, as well as the ratio of whole-body extracellular to intracellular fluid (ECF/ICF) were assessed. As we did not measure the weight of the lower limbs, limb extracellular (limbECF) as well as intracellular fluid (limbICF) were calculated from the segmental measurement, based on the limb length, limb circumferences, extracellular impedance (Ri), intracellular impedance (R0) and specific resistances (for gender). The ratio between limb extracellular and intracellular fluid (limbECF/limbICF) was calculated, and expressed as the ratio of intracellular impedance (Ri) to extracellular impedance (R0) [20,22].

### 2.6. Assessment of Plasma Volume Changes

Three different equations were used to calculate relative plasma volume changes (%) pre and post MLD based on a combination different plasma parameters: (1) plasma density (PD) and hematocrit (Hct) [23], (2) hematocrit (equation according to van Beaumont [24]) or (3) hematocrit and hemoglobin (Hb) (equation according to Dill and Costill [25]). Additionally, the formula according to Nadler (4) was used to estimate the absolute values of plasma volume changes [26].

All blood parameters assessed in this study were corrected to plasma volume changes according to (2) the equation according to Van Beaumont [24], as Hct as well as Hb perfectly matched after correction using the following equation (*Hct_B_* represents the baseline value, whereas *Hct_A_* represents the value post-MLD):

$$\%\Delta PV=\frac{100*(Hct_{B}-Hct_{A})}{Hct_{A}*(100-Hct_{B})}$$

### 2.7. Statistics

Data are presented as mean ± standard deviation (SD). Analysis of variances (two-way) ANOVA with repeated measurements was conducted in order to determine effects over three weeks of lymphedema therapy and due to manual lymphatic drainage averaged over three weeks. Correlation was tested using Pearson’s correlation with Fisher z-transformation to calculate a mean correlation over all time-points. *p*-values < 0.05 were considered as statistically significant. All statistical analyses were performed using IBM SPSS (v26.0, SPSS Inc., Chicago, IL, USA) software. GraphPad Prism (v8.4.1, GraphPad Software, Inc., San Diego, CA, USA) was used to generate the figures. Figures display mean values ± standard error of the mean (SEM).

## 3. Results

In total, thirteen patients (3 males and 10 females, 57 ± 8.0 years of age, 167.2 ± 8.3 cm height, 91.0 ± 23.4 kg weight) with stage II primary or secondary lymphedema were included in this study. Out of these patients, 10 were diagnosed with bilateral lower limb lymphedema, whereas 3 were diagnosed with unilateral lymphedema. All patients completed the study protocol.

### 3.1. Reduction of Lower Limb Volume Due to Physical Therapy Assessed via Perometry

**Figure 2:**
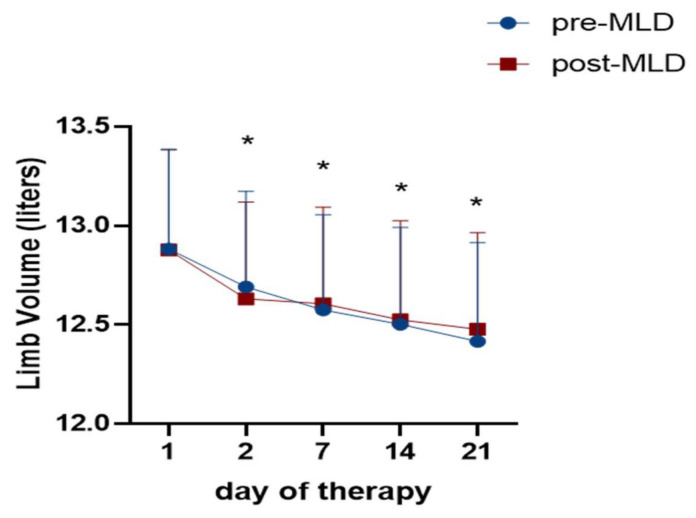
Time course of lower limb volume (liters) assessed by perometry over three weeks of complete decongestive therapy (CDT), before (**blue dots**) and after (**red boxes**) manual lymphatic drainage (MLD). Asterisks (*) indicate a significant decrease of limb volume over three weeks of therapy (*p* < 0.001) compared to baseline.

As expected, leg volume decreased over three weeks of therapy from 12.840 ± 1.899 L to 12.448 ± 1.977 L (F_(2;37)_ = 31; *p* < 0.001; ε = 0.74). This is a total loss of 0.392 L or 3.1% (Figure 2). The major reduction occurred between the first (12.840 ± 1.899 L) and second (12.640 ± 1.820 L) day of CDT with a volume reduction of 1.5% (t_(17)_ = 4.98; *p* = 0.0001). No changes in leg volume were seen pre vs. post MLD (F_(1;15)_ = 0.42; *p* = 0.527).

### 3.2. Changes in Lower Limb Fluid Due to Physical Therapy

**Figure 3:**
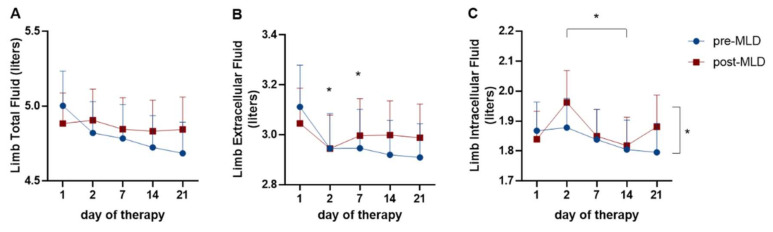
Time course of (**A**) total fluid, (**B**) extracellular fluid and (**C**) intracellular fluid changes in the lower limbs over three weeks of complete decongestive therapy (CDT) and due to manual lymphatic drainage (MLD). The blue circles represent values pre-MLD, the red boxes indicate fluid concentrations post-MLD. Asterisks (*) represent significant differences (*p* < 0.05).

Extracellular limb volume (limbECF) reduced over three weeks of CDT (F_(4;76)_ = 2.60; *p* = 0.041). The highest reduction was seen between day 1 (3.068 ± 0.640 L) and day 2 (2.795 ± 0.715 L, t_(__25)_ = 3.03; *p* = 0.006) as well as day 7 (2.905 ± 0.663 L, t_(__25)_ = 2.75; *p* = 0.011) of CDT (Figure 3B). LimbECF did not change due to MLD (F_(1;19)_ = 1.04; *p* = 0.321).

Intracellular fluid in the limbs (limbICF) decreased over three weeks (F_(4;76)_ = 4.11; *p* = 0.005) and increased post-MLD (F_(1;19)_ = 6.85; *p* = 0.017). LimbICF was significantly different between day 2 (1.920 ± 0.453 L) and day 14 (1.811 ± 0.430 L) of therapy (t_(__19)_ = 3.43; *p* = 0.003). MLD led to an average increase in limbICF from 1.837 ± 0.037 L pre-MLD to 1.879 ± 0.056 L post-MLD, which reflects an increase of 2.3% (Figure 3C).

LimbTBW tended to reduce over three weeks of CDT from 4.987 ± 0.910 L to 4.824 ± 0.926 L (F_(3; 51)_ = 2.77; *p* = 0.057; ε = 0.79) and did not change due to MLD (F_(1;19)_ = 3.05; *p* = 0.097) (Figure 3A).

**Figure 4:**
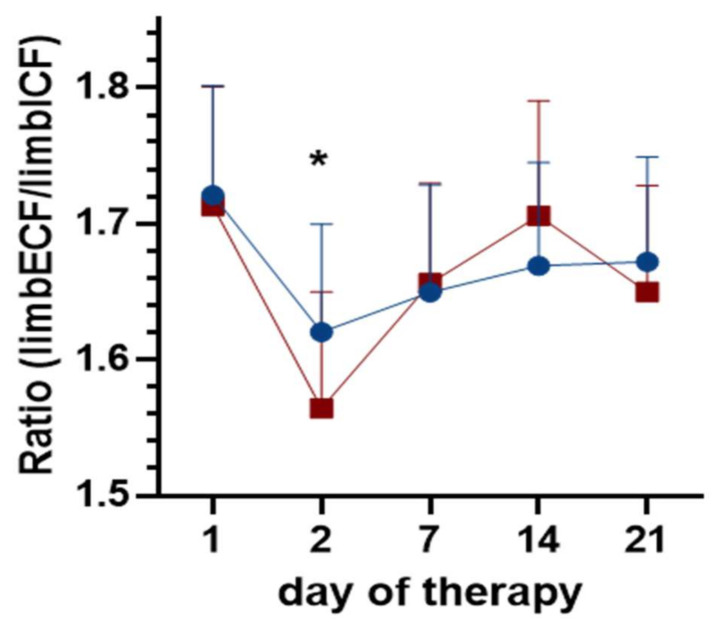
Time course of limb extracellular fluid normalized to limb intracellular fluid (ratio) over three weeks of complete decongestive therapy (CDT) and due to manual lymphatic drainage (MLD). The **blue circles** represent values pre-MLD, the **red boxes** indicate fluid concentrations post-MLD. Asterisks (*) represent significant differences (*p* < 0.05).

Ratio of limbECF normalized to limbICF (limbECF/limbICF) significantly reduced over three weeks of CDT (F_(4;76)_ = 4.49; *p* = 0.003), but did not change due to MLD (F_(1;19)_ = 0.28; *p* = 0.601). Post-test showed a major reduction between day 1 and day 2 of CDT (*p* = 0.003) (Figure 4).

### 3.3. Correlation Between Perometry and BIS

Perometry and limbTBW moderately correlated over all measurement time points (*r* = 0.635).

### 3.4. Whole-Body Fluid Shifts Measured by Bioelectrical Impedance Spectroscopy

**Figure 5:**
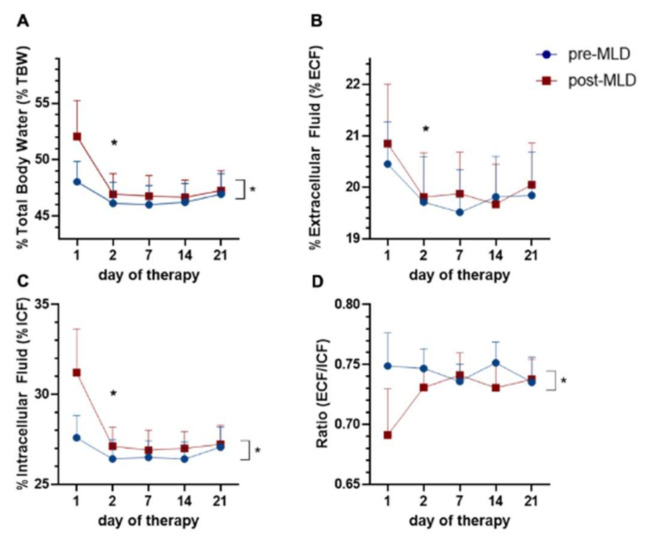
Time course of percental changes, of (**A**) total body water (%TBW), (**B**) extracellular fluid (%ECF), (**C**) intracellular fluid (%ICF), to the individual patient’s body weight, as well as (**D**) extracellular fluid normalized to intracellular fluid (ECF/ICF) over three weeks of complete decongestive therapy (CDT) and due to manual lymphatic drainage (MLD). The blue circles represent values pre-MLD, the red boxes indicate fluid concentrations post-MLD. Asterisks (*) represent significant differences (*p* < 0.05).

Percentage of total body water (%TBW) reduced over three weeks of therapy (F_(4;28)_ = 7.04; *p* = 0.001) and increased post-MLD (F_(1;7)_ = 11.84; *p* = 0.011). The major reduction of %TBW occurred between the first and second day of CDT (t_(13)_ = 3.09; *p* = 0.009), with a reduction from 50% to 46.5%. Overall reduction over three weeks of CDT was from 50% to 47% total body water. The average increase in %TBW due to MLD was from 46% pre-MLD to 48% post-MLD (Figure 5A).

Percentage of extracellular fluid (%ECF) decreased over three weeks of CDT (F_(4;40)_ = 4.15; *p* = 0.007), with the highest decrease between day 1 and day 2 (t_(13)_ = 2.48; *p* = 0.029). Overall reduction was 0.8% (from 20.7% to 19.9%) in whole body extracellular fluid. MLD did not lead to changes in %ECF (F_(1;10)_ = 2.17; *p* = 0.172) (Figure 5B).

Percentage of intracellular fluid (%ICF) increased post-MLD (F_(1;10)_ = 15; *p* = 0.003). This represents an increase of 1 percentage point. No changes in %ICF were seen over three weeks of CDT (F_(1;12)_ = 4.04; *p* = 0.063) (Figure 5C).

Ratio of ECF/ICF significantly changed due to MLD over three weeks of CDT (F_(1;10)_ = 8.97; *p* = 0.013). No changes occurred due to three weeks of CDT (F_(1;14)_ = 0.56, *p* = 0.521) (Figure 5D).

### 3.5. Plasma Volume Changes (PVC) Due to Physical Therapy

**Figure 6:**
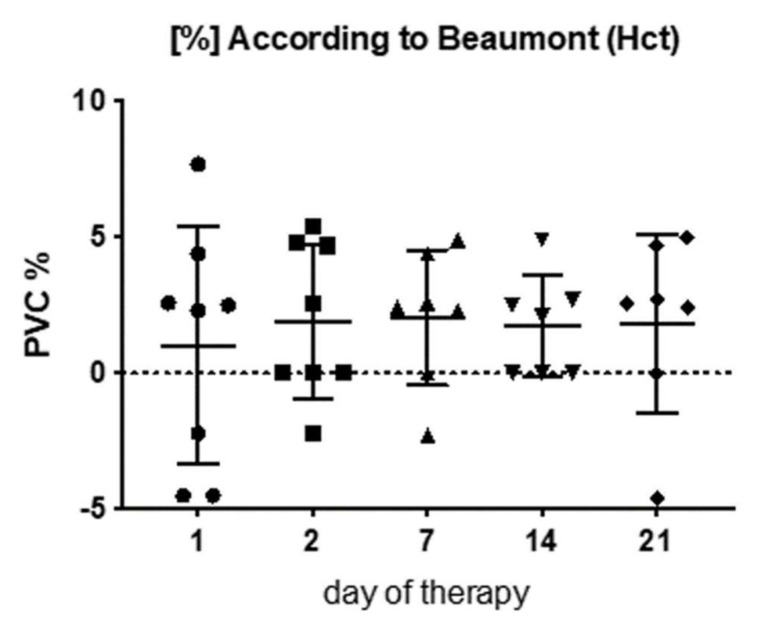
Plasma volume changes (PVC) calculated from hematocrit variations according to van Beaumont [24].

All the methods and equations for plasma volume changes calculations led to similar results. Plasma volume increased post-MLD, in average by 1.5 ± 0.8%, but did not change over 3 weeks of therapy (F_(2;24)_ = 0.88, *p* = 0.489) (Figure 6).

### 3.6. Plasma Protein Changes Due to Physical Therapy

**Figure 7:**
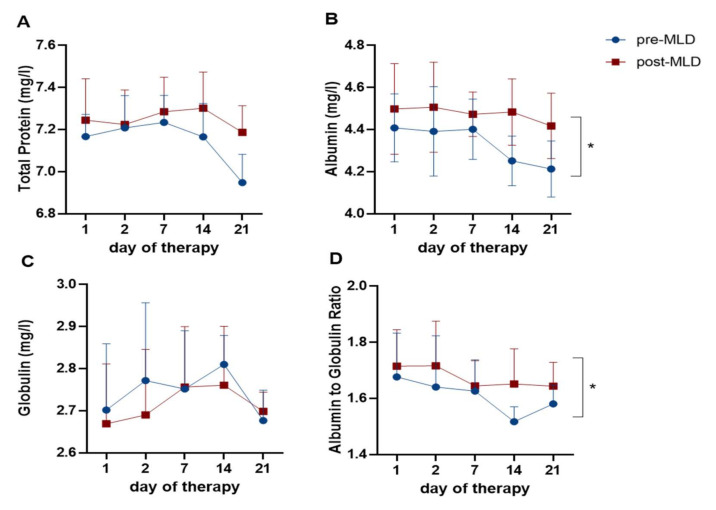
Time-course of changes in (**A**) total protein (mg/L), (**B**) albumin (mg/L), (**C**) globulin (mg/l) and (**D**) albumin-to-globulin ratio (A/G) over three weeks of complete decongestive therapy (CDT) and due to manual lymphatic drainage (MLD). The blue circles represent values pre-MLD, the red boxes indicate fluid concentrations post-MLD. Asterisks (*) indicate significant changes in albumin (*p* = 0.005) and A/G ratio (*p* = 0.049) pre-compared to post-MLD.

Albumin, as well as albumin-to-globulin ratio (A/G ratio), increased after MLD (F_(1;5)_ = 23.71; *p* = 0.005 and F_(1;5)_ = 6.63; *p* = 0.049, respectively) from 4.33 ± 0.09 to 4.47 ± 0.03 mg/L and 1.61 ± 0.06 to 1.67 ± 0.04 mg/L, which is an increase of 3.3% and 4.1%, respectively (Figure 7). Total plasma protein levels tended to increase post-MLD (F_(1;6)_ = 5.04; *p* = 0.066). Total protein concentrations did not change over three weeks of therapy (F_(4;24)_ = 0.96; *p* = 0.445) (Figure 7A).

### 3.7. Plasma Osmolality and Oncotic Pressure

Plasma osmolality and oncotic pressure did not change over three weeks of CDT (F_(4;12)_ = 1.01; *p* = 0.440; ε = 0.48 and F_(1;6)_ = 1.02; *p* = 0.366; ε = 0.31, respectively) or due to MLD (F_(1;3)_ = 2.14; *p* = 0.239 and F_(1;5)_ = 0.08; *p* = 0.789, respectively).

### 3.8. Electrolyte Concentrations

Plasma electrolyte levels showed an interaction between therapy days and MLD (*Sodium*: F_(4;16)_ = 4.35; *p* = 0.014; *Chloride*: F_(4;16)_ = 4.35; *p* = 0.014; *Potassium*: F_(4;12)_ = 4.62; *p* = 0.011). Sodium, chloride and potassium levels reduced post-MLD on day one, but increased on all other measurement days compared to pre-MLD. Electrolyte levels did not change over three weeks of CDT (*Sodium*: F_(4;16)_ = 0.86; *p* = 0.509; *Chloride*: F_(1;16)_ = 1.10; *p* = 0.389; *Potassium*: F_(4;12)_ = 0.92; *p* = 0.474). Finally, no changes were seen due to MLD (*Sodium*: F_(1;4)_ = 1.00; *p* = 0.374; *Chloride*: F_(1;4)_ = 0.35; *p* = 0.585; *Potassium*: F_(1;3)_ = 0.06; *p* = 0.824).

## 4. Discussion

We observed the expected decrease in limb volume over three weeks of complete decongestive therapy. Extracellular—and intracellular—fluid in the limbs decreased over time, whereas total leg fluid only tended to reduce due to three weeks of lymphedema therapy (*p* = 0.057). Intracellular limb fluid increased due to manual lymphatic drainage. A moderate correlation was observed between total leg volume (assessed via perometry) and total leg fluid (assessed via bioelectrical impedance spectroscopy). Whole-body fluid assessment showed a decrease in total body—and extracellular—fluid over three weeks of therapy and an increase after manual lymphatic drainage. Our results suggest that a major fluid change occurred between the first and second day of lymphedema therapy for limb extracellular fluid (*p* = 0.006), whole-body total fluid (*p* = 0.009) and extracellular fluid (*p* = 0.002). We further observed an increase in plasma volume due to manual lymphatic drainage of, on average, 1.5%. Albumin concentrations as well as albumin-to-globulin ratio increased post lymphatic drainage.

### 4.1. Reduction of Lower Limb Volume Due to Physical Therapy

Our results show an overall reduction of leg volume of 392 mL (3.1%) over three weeks of complete decongestive therapy (*p* < 0.001). Cohen and colleagues (2011) reported a reduction of 368 mL post-therapy in a case study. The therapy protocol used in their study included eight sessions over three months [27]. Compared to the study by Cohen (2011), the treatment protocol used in our study was shorter, but led to a similar outcome. This could be due to the higher frequency of therapy sessions (daily manual lymphatic drainage) used in this study. In another study, performed by Kostanoğlu and colleagues (2019), a reduction between 296 mL and 1038 mL, depending on the stage of lymphedema, was reported after 4 weeks of complete decongestive therapy [7]. A recent study by Cavezzi and colleagues (2020) also showed a short-term reduction of limb volume using perometry after 6 days of complete decongestive therapy [28]. We observed similar effects in our study after 21 days.

Leg volume, however, did not change due to manual lymphatic drainage (*p* = 0.527). This is a novel finding of our study, as in the previous studies, perometry was assessed before and after several weeks of physical therapy, but not directly before and after manual lymphatic drainage [7,29,30,31]. A recent study by Habnouni and colleagues (2020) reported that 30 min of manual lymphatic drainage led to a reduced limb circumference in children with lower limb lymphedema, however, limb volume did not decrease [32]. This is in agreement with our results. These findings suggest that perometry may not be sensitive enough to detect short-term fluid shifts induced by physical therapy in lower limb lymphedema patients.

### 4.2. Changes in Lower Limb Fluid Due to Physical Therapy

Over three weeks of complete decongestive therapy, extracellular—as well as intracellular—fluid reduced in the lower limbs. This is also reflected in a reduction in the inter-leg ratio of extracellular—to intracellular—fluid (expressed as the ratio of Ri to R0) over three weeks of physical therapy. We also observed a tendency towards a reduced total limb fluid (limbTBW) after three weeks of physical therapy (*p* = 0.057). Different studies have previously investigated extracellular and intracellular fluid shifts during lymphedema therapy via bioelectrical impedance spectroscopy [33,34,35]. De Godoy and colleagues (2013) assessed fluid shifts before—and after—one week of intensive physical treatment in lower limb lymphedema patients [16]. They found a significant reduction of total, extracellular and intracellular limb fluid in the affected body part, whereas total water increased in the healthy body parts (upper limbs and trunk). They concluded that fluid is mobilized from the affected body parts to the healthy ones [16]. Pareira de Godoy and colleagues (2019) obtained similar results: post-therapy induced reduction in intra- and extracellular water in the limbs [36]. As with the study by Pareira de Godoy and colleagues (2019), we also obtained significant reduction of limbECF as well as limbECF/limbICF ratio within the first week of CDT (that is, baseline values as compared to those of day 2 and day 7).

Yamamoto and colleagues (2008) observed a volume reduction of 55% from day 1 to day 2 of therapy, a volume reduction of 11% and 1% between day 2 to 3 and day 4 to day 6, respectively [37]. Our results are in accordance with the findings of Yamamoto and colleagues (2008), confirming that the biggest effect of physical therapy occurs within the first week of therapy. This indicates that the body is able to adapt to perturbations rather rapidly (e.g., within one week) and that no significant changes occur afterwards. Similar observations have previously been reported, for example, during space flight or in bed rest studies [38,39,40,41,42].

We tested the hypothesis that extracellular fluid in the limbs (limbECF) decreases after manual lymphatic drainage (as lymphatic drainage should enhance lymphatic outflow), and that the limb intracellular fluid will not change. Our data disprove this hypothesis, as manual lymphatic drainage in the limbs did not lead to extracellular fluid changes. In accordance with this observation, no changes were seen in limbECF/limbICF ratio due to MLD. Only intracellular limb fluid increased (*p* = 0.017). This is in accordance with the results of a study performed by Maher and colleagues (2012). They did also not observe changes in limbECF after manual lymphatic drainage in arm lymphedema patients [43]. Although we have not studied individual components of complete decongestive therapy separately, our results show that complete decongestive therapy leads to fluid shifts, whereas manual lymphatic drainage alone is not sufficient enough to influence segmental fluid shifts (assessed directly before and after lymphatic drainage). This is in accordance with previous reports suggesting that manual lymphatic drainage does not add further benefits to complete decongestive therapy [44,45,46], especially in moderate to severe cases [47].

### 4.3. Correlation between Perometry and Bioelectrical Impedance Spectroscopy

Our results showed a moderate correlation (r = 0.635) between perometry (assessing leg volume) and bioelectrical impedance spectroscopy analysis (assessing leg fluid) at all measurement timepoints. Previously, Jain and colleagues (2010) reported that bioelectrical impedance spectroscopy produces valid and reliable data as compared to the gold standard assessment, which is perometry [48]. However, in their study, only cancer-related arm lymphedema patients were included. Similar to this, a study by Bundred and colleagues (2015) showed a moderate correlation of perometry and bioelectrical impedance spectroscopy in early detection of arm lymphedema in cancer patients [49]. To our knowledge, correlations between these two methods for the assessment of physical therapy outcome [31] have not yet been investigated in patients with lower limb lymphedema.

### 4.4. Whole-Body Fluid Shifts Due to Lymphedema Therapy

Another hypothesis that we tested was that whole-body total and extracellular fluid decreases following three weeks of lymphedema therapy. We observed decreases in total body water (%TBW) and extracellular fluid (%ECF) after three weeks of therapy, which is in accordance with our proposed hypothesis. Similar to what we observed in the segmental analysis, the greatest fluid reduction in the whole-body assessment occurred between day 1 and day 2 of therapy (%TBW: t_(13)_ = 3.09; *p* = 0.009 and %ECF: t_(13)_ = 2.48; *p* = 0.029). Our results indicate that in terms of fluid mobilization, one week of therapy may be enough, as no changes occur afterwards.

In addition, our results suggest that whole-body intracellular fluid (%ICF) changes over the three weeks of therapy. Pereira de Godoy and colleagues (2019) reported increases in intracellular fluid after 7 days of intensive therapy. In contrast to that, our results show a reduction of intracellular fluid over three weeks of therapy, but an increase of intracellular fluid due to manual lymphatic drainage. The differences in our findings could be attributed to the methodology used: they assessed only the trunk and upper abdomen [36], whereas we assessed overall body fluids.

Furthermore, whole-body ratio of extracellular to intracellular fluid (ECF/ICF) decreases due to manual lymphatic drainage, averaged over three weeks of physical therapy. This is in contrast with what has been reported. For instance, a recent review by Thompson and colleagues (2020) reported that in further stages of lymphedema (stage II–III) manual lymphatic drainage may not provide an additional benefit in combination with complex decongestive therapy [47]. While this might be true for changes in limb fluid in our study, our novel results show that manual lymphatic drainage on its own can lead to changes in whole-body fluids.

### 4.5. Plasma Volume and Plasma Component Changes Due to Fluid Shifts During Lymphedema Therapy

Manual lymphatic drainage has been suggested to enhance lymphatic activity in healthy patients [50] and to improve transport of radiotracers in the lymphatic system in lymphedema patients [51]. We tested the hypothesis that the fluid shifts due to physical therapy would also be reflected in plasma volume changes, and could be used as indicator for lymphatic outflow. Our results show that lymphatic drainage leads to an average increase in plasma volume of 1.5 ± 0.8%. The plasma volume increases we observed post-therapy suggest that lymphatic fluid is entering the blood stream. Plasma volume increase/expansion can be typically seen during pregnancy, exercise or heat exposure [52]. Exercise, for instance, can lead to an average plasma increase of 9–15% [53]. Plasma volume changes due to manual lymphatic drainage in our study reached lower levels (1.5%) than seen during exercise. To our knowledge, no previous study has investigated short-term plasma volume changes due to physical therapy. Increased plasma volume status (a person’s deviation from their ideal plasma volume level) has previously been associated with an increased mortality from cardiovascular diseases in the general population [54].

In contrast to that we expected, albumin levels increased after manual lymphatic drainage, which is also reflected in changes in the albumin-to-globulin ratio. Plasma volume changes have previously been reported to affect plasma protein concentrations [18,55]. However, this was investigated in perturbations of physiological states that are associated with plasma volume losses (e.g., after presyncope or postural changes) and not plasma volume increases, as occurs following lymphatic drainage. As albumin concentrations were corrected to plasma volume changes—and as hematocrit did not change due to manual lymphatic drainage—increases in albumin could not have occurred due to the only fluid shifts. Therefore, our results indicate that the excess fluid entering the blood stream (that is, the lymphatic fluid mobilized due to the therapy) has a higher albumin content than plasma. To our knowledge, this has not been previously reported.

Due to the increased plasma albumin levels that we observed post-therapy, we also expected accompanying changes in oncotic pressure. However, we did not observe changes in the oncotic pressure. Furthermore, concentrations of electrolytes such as sodium, chloride and potassium did not change over three weeks of therapy. A possible reason could be the relatively small increase in plasma volume (approximately 1.5%). In accordance with these observations, osmolality did not show any changes as well. Our results further showed that on the first day of therapy, osmolality and electrolyte levels decrease post-MLD, whereas they increase on all other measurement time-points. Therefore, significance was not achieved comparing pre- vs. post-MLD. This could be because the greatest changes in fluid mobilization occurred between the first and second day of CDT.

Overall, our results suggest that the equation according to Van Beaumont is a suitable tool to assess minor plasma volume changes, as they occur due to fluid shifts during lymphedema therapy. Although our results indicate that fluid entering the blood stream has a higher protein content than plasma, it seems as if manual lymphatic drainage does not lead to major changes in plasma components (e.g.,: electrolytes) in our patients. Future studies should investigate if other plasma components such as hormones and blood cells are affected by physical therapy and if plasma volume and plasma component analysis could provide a promising and cost-effective method to assess lymphatic (out-)flow as well as lymphatic fluid concentration in different lymphedema patient groups (e.g., different grades) [56]. 

### 4.6. Limitations

A possible limitation of this study could be that data sets of all lower limbs were pooled. Therefore, we did not calculate ratios between affected and non-affected lower extremities. The reason for this is that the majority of the patients included in this study were diagnosed with bilateral lower leg lymphedema and the number of non-affected legs available for analysis was limited (*n* = 3). We do not, however, believe that this is a limitation, as exclusion of the non-affected legs did not influence our results.

Another limitation could be that patients with both primary and secondary lymphedema were included. Although the underlying mechanisms of primary and secondary lymphedema are different [57], the therapeutic approach is identical [11]. Other studies, which investigated effects of lymphedema therapy, did not distinguish between primary and secondary lymphedema as well [7,15]. Noh and colleagues (2015), for example, did not find any differences between primary and secondary lymphedema in therapy outcome (leg volume post-therapy) [58].

Finally, more females than males were enrolled in our study. However, we do not think this is a limitation, as more females than males are affected by this progressive and debilitating lymphatic disease [59].

## 5. Conclusions

Our results indicated fluid shifts occurring due to physical therapy and manual lymphatic drainage. The greatest fluid shift in lymphedema patients occurred within the first week of complete decongestive therapy. Manual lymphatic drainage affects the limbs and whole-body fluid composition. Although perometry and bioelectrical impedance spectroscopy showed a moderate correlation, perometry does not appear sensitive enough to detect small fluid changes as they occur due to manual lymphatic drainage.

Fluid shifts due to physical therapy were also reflected in increased plasma volume and plasma protein concentrations after manual lymphatic drainage. This could be an indirect indicator of the concentration and composition of lymphatic fluid entering into the blood stream through the thoracic duct. As plasma volume increases due to physical therapy could be associated with compensatory hemodynamic and volume regulatory hormonal responses, these aspects should be examined in future studies.

Future studies should further investigate how fluid shifts induced by lymphedema therapy are modulated by disease etiology (primary or secondary lymphedema), affected body part (upper and/or lower limbs) and different therapeutical approaches.

## Abbreviations

BIS, Bioelectrical impedance spectroscopy; CDT, Complete decongestive therapy; ECF, Extracellular fluid; Hct, Hematocrit; ICF, Intracellular fluid; limbICF, Limb intracellular fluid; limbECF, Limb extracellular fluid; limbTBW, Total limb fluid; MLD, Manual lymphatic drainage; PD: Plasma density; TBW, Total body water.
